# MicroRNAs and Drinking: Association between the Pre-miR-27a rs895819 Polymorphism and Alcohol Consumption in a Mediterranean Population

**DOI:** 10.3390/ijms17081338

**Published:** 2016-08-16

**Authors:** Rocío Barragán, Oscar Coltell, Eva M. Asensio, Francesc Francés, José V. Sorlí, Ramon Estruch, Albert Salas-Huetos, Jose M. Ordovas, Dolores Corella

**Affiliations:** 1 Department of Preventive Medicine and Public Health, School of Medicine, University of Valencia, Valencia 46010, Spain; rocio.barragan@uv.es (R.B.); eva.m.asensio@uv.es (E.M.A.); francesc.frances@uv.es (F.F.); jose.sorli@uv.es (J.V.S.); 2 CIBER Fisiopatología de la Obesidad y Nutrición, Instituto de Salud Carlos III, Madrid 28029, Spain; oscar.coltell@uji.es (O.C.); RESTRUCH@clinic.cat (R.E.); albert.salas@uab.cat (A.S.-H.); 3 Department of Computer Languages and Systems, School of Technology and Experimental Sciences, Universitat Jaume I, Castellón 12071, Spain; 4 Department of Internal Medicine, Hospital Clinic, IDIBAPS, Barcelona 08036, Spain; 5 Human Nutrition Unit, Biochemistry and Biotechnology Department, IISPV, University Rovira i Virgili, Reus 43003, Spain; 6 Department of Cardiovascular Epidemiology and Population Genetics, Centro Nacional de Investigaciones Cardiovasculares (CNIC), Madrid 28029, Spain; jose.ordovas@tufts.edu; 7 IMDEA Alimentación, Madrid 28049, Spain; 8 Nutrition and Genomics Laboratory, JM-USDA Human Nutrition Research Center on Aging at Tufts University, Boston, MA 02111, USA

**Keywords:** microRNAs, alcohol, miR27a, Mediterranean

## Abstract

Recently, microRNAs (miRNA) have been proposed as regulators in the different processes involved in alcohol intake, and differences have been found in the miRNA expression profile in alcoholics. However, no study has focused on analyzing polymorphisms in genes encoding miRNAs and daily alcohol consumption at the population level. Our aim was to investigate the association between a functional polymorphism in the pre-miR-27a (rs895819 A>G) gene and alcohol consumption in an elderly population. We undertook a cross-sectional study of PREvención con DIeta MEDiterránea (PREDIMED)-Valencia participants (*n* = 1007, including men and women aged 67 ± 7 years) and measured their alcohol consumption (total and alcoholic beverages) through a validated questionnaire. We found a strong association between the pre-miR-27a polymorphism and total alcohol intake, this being higher in GG subjects (5.2 ± 0.4 in AA, 5.9 ± 0.5 in AG and 9.1 ± 1.8 g/day in GG; *p*_adjusted_ = 0.019). We also found a statistically-significant association of the pre-miR-27a polymorphism with the risk of having a high alcohol intake (>2 drinks/day in men and >1 in women): 5.9% in AA versus 17.5% in GG; *p*_adjusted_ < 0.001. In the sensitivity analysis, this association was homogeneous for sex, obesity and Mediterranean diet adherence. In conclusion, we report for the first time a significant association between a miRNA polymorphism (rs895819) and daily alcohol consumption.

## 1. Introduction

In recent years, multiple and important regulatory functions have been attributed to microRNAs (mirRNA) [1]. It is known that miRNAs, small noncoding RNAs, whose final product is a ~22-nucleotide functional RNA molecule that regulates gene expression, play an important role in processes, such as controlling oxidative stress, the development, progression and metastasis of cancer, influence the processes of atherosclerosis, cardiovascular diseases, obesity and diabetes and control senescence and many other key processes [1,2,3,4,5]. Although it has been suggested that the miRNA may also play an important role in influencing food and beverage intake [6,7,8], there have been far fewer studies on this, and further studies that delve deeper into this issue are required. Among the different foods and beverages, where there is indeed more data suggesting an important regulation by microRNAs, is alcohol intake [8].

The association between moderate alcohol consumption and health is a matter of on-going debate in the scientific community [1,3,9,10]. Likewise, there is a debate over the factors that influence alcohol intake, these being attributed both to environmental factors (socioeconomic level, one’s relatives’ alcohol consumption behavior, one’s social network, social myths and violence, among others) and genetic (variations in candidate genes), without their contribution having been clearly quantified [7,8,11]. Among the genetic factors, as is the case with environmental factors, there appears to be a huge complexity of influences that are still not well understood [7,8,11,12,13,14,15]. Although the first studies on the influence of alcohol intake specifically analyzed genetic variants in candidate genes related to the different pathways on which alcohol acts or is metabolized (mainly in polymorphisms in genes related to alcohol-metabolizing enzymes, including: alcohol dehydrogenase (*ADH*) and aldehyde dehydrogenase (*ALDH*), where important associations have been found) [11,12], it is known that there are many other factors to be investigated.

Indeed, a new line of research into the genetic-epigenetic factors that may have an influence on alcohol intake involving regulation by miRNAs is emerging [8,16]. miRNAs are highly abundant in the brain and play significant roles in several biological processes [17]. Accordingly, it has been reported that miRNAs also seem to mediate the cellular adaptations induced by exposure to some drugs of abuse [18], including cocaine [19], opioids [20] and alcohol [21,22,23]. Moreover, Gedik et al. [24] hypothesized that single nucleotide polymorphisms (SNPs) in the miRNA biogenesis pathway may result in dysregulation of miRNA levels and association with alcohol dependence. Consequently, they found statistically-significant associations between several SNPs in the miRNA biogenesis and alcohol dependence when alcohol-dependent patients were compared to healthy controls. The SNPs analyzed were: rs595961, rs4961280, rs910924 and rs1640299 in the genes *AGO1* (Argonaute 1), *AGO2* (Argonaute 2), *GEMIN4* (gem nuclear organelle-associated protein 4), *DGCR8* (DiGeorge syndrome critical region 8 complex subunit), respectively; adding more evidence to the role of microRNAs in alcohol intake.

However, although there have been various studies that have analyzed the differences of miRNA expression associated with differing conditions of alcohol consumption, statistically-significant differences being found between the micro-RNA profile of alcoholics compared to non-alcoholics [19,21,22,23,25,26,27,28], there are hardly any that have investigated the association between SNPs in genes that encode miRNAs and alcohol intake [29].

miRNA genes are transcribed and processed initially into precursor miRNAs (pre-miRNAs). The pre-miRNAs are further processed into mature miRNAs. SNPs in the pre-miRNA genes could affect the processing and subsequent maturing of miRNAs. Interestingly, it has been reported that the occurrence of SNPs in miRNA sequences is relatively rare [30], suggesting that variation in the miRNA sequence might be functionally important. There are various miRNA candidates for which the study of the effects of their sequence SNPs on alcohol intake would be interesting. We first focused our attention on the pre-miR-27a rs895819 A>G polymorphism, located on the terminal loop of the miR-27a, because some recent studies have related this miRNA with alcohol modulation in different processes [31,32], as well as with the behavioral response to chronic opioid administration [33]. Moreover, there are previous studies that have shown that that polymorphism is functional, an increased miR-27a expression being detected in G-allele carriers compared to AA [26,27,34,35]. In parallel, this polymorphism has been associated with increased risk of some cancers related to alcohol consumption (gastric, colorectal, etc.) G-allele carriers [26,27,28,34,35,36]. Although a recent study has found that the plasma of alcoholic patients has an increased number of extracellular vesicles that contained high levels of miR-27a compared to healthy controls [31], no study has investigated the association between the pre-miR-27a rs895819 SNP and alcohol consumption. What is more, as far as we know, there has been only one study published to date that has examined the association between a miRNA SNP and alcohol [29], but this analyzed the prevalence of alcohol-related disorders without investigating habitual alcohol intake on the population level. Thus, given this lack of studies, our aim was to analyze the association between the pre-miR-27a rs895819 polymorphism and total alcohol intake, as well as the different alcoholic drinks consumed (wine, beer, spirits) in a well-characterized elderly Mediterranean population, recruited in one of the centers participating in the PREDIMED (PREvención con DIeta MEDiterránea) study [37].

## 2. Results

The participants analyzed in this study were all recruited at the PREDIMED-Valencia field center [38], one of the centers participating in the PREDIMED multicenter study [37]. The PREDIMED-Valencia study is where most patients have been recruited and randomized (*n* = 1094) for the PREDIMED study. Figure S1 presents a flow chart of the study participants. We analyzed 1007 men and women who had valid genotypes for the pre-miR-27a rs895819 A>G polymorphism and their alcohol consumption determined. Table 1 provides an overview of the population distribution of the demographic, clinical, biochemical and lifestyle characteristics of the 1007 participants according to the pre-miR-27a rs895819 A>G polymorphism. Prevalence of the genotypes was 53.6% AA (*n* = 540); 37.8% AG (*n* = 381) and 8.5% GG (*n* = 86), similar to the prevalence expected for European populations. The mean age of the study participants (mean ± SE) was 66.8 ± 0.2 years and did not differ among the pre-miR-27a rs895819 genotypes. There were also no statistically-significant differences in sex, type 2 diabetes prevalence, weight-related variables, blood pressure, lipid levels, fasting glucose, smoking, physical activity, total energy intake, macronutrients (fat, carbohydrates and proteins) or adherence to the Mediterranean diet (MedDiet) between the genotypes, so minimizing the bias that these factors may have on the association between the SNP and alcohol consumption.

### 2.1. Association between the Pre-miR-27a rs895819 Polymorphism and Total Alcohol Consumption and Types of Alcoholic Beverages

We analyzed the association between the pre-miR-27a rs895819 polymorphism and total alcohol intake, as well as with alcoholic beverages in the population as a whole. We measured the intake of alcoholic beverages (different types of wine, beer and spirits) by a validated [39] food frequency questionnaire (FFQ) as detailed in the Methods. In this FFQ, there were questions about the average intake for each beverage over the previous year, including the baseline visit, which is when the questionnaire was administered. Alcohol intake (g/day) was calculated by multiplying the amount of the corresponding beverage in the FFQ (mL/day) by the respective alcohol content (see the Methods in Section 4.2). The sum of all of that is the total amount of alcohol in g/day consumed for each person. Abstainers were those individuals for whom the sum of alcohol consumption was zero grams per day. For the analysis of specific beverages, total wine, total beer and total spirits were first considered (see the Methods in Section 4.2). That does not imply that a person who drinks beer does not also drink wine or spirits. When the association between the pre-miR-27a rs895819 polymorphism with total alcohol intake and alcoholic beverages was analyzed (Table 2), we obtained statistically-significant results. Total alcohol consumption was considered as a continuous variable, and men and women were analyzed together. The average alcohol consumption (and standard error) for the whole population was 5.8 (0.3) g/day. Taking into account that the analyzed population was an elderly population not including alcoholics (see the Methods in Section 4.1), the mean intake of alcoholic beverages was relatively low.

We observed a statistically-significant association between the pre-miR-27a rs895819 polymorphism and total alcohol intake (g/day). Total alcohol consumption in carriers of the variant G-allele was higher than in the other genotypes: 5.2 (0.3) g/day in AA; 5.9 (0.5) g/day in AG and 9.1 (1.8) g/day in GG, *p* = 0.020 in the unadjusted general linear model (GLM) for a linear trend. This association remained statistically significant even after multivariable adjustment for sex, age, type 2 diabetes, hypertension, dyslipidemia, obesity, smoking, physical activity and total energy intake (*p* = 0.016) in the adjusted model (GLM). Alcohol intake had a skewed distribution and required normalization for statistical testing. Then, although means of alcohol intake were shown as untransformed variables, *p*-values were computed using the square root-transformed variables for total alcohol intake, as well as for alcoholic beverages, to improve normality.

**Figure 1:**
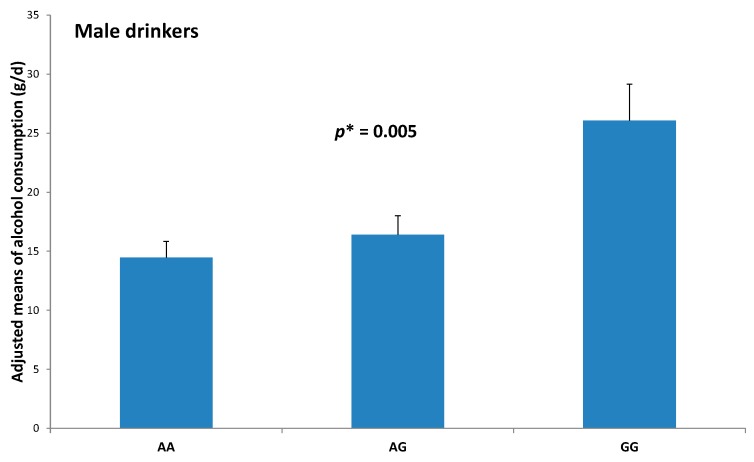
Adjusted means of total alcohol intake (g/day) in drinker males depending on pre-miR-27a rs895819 polymorphism. Means and standard errors (SE) were estimated by the multivariable general linear model adjusted for age, type 2 diabetes, obesity, hypertension, dyslipidemia, physical activity, smoking and total energy intake. For statistical significance, the transformed (square root) alcohol consumption variable was used. AA (*n* = 148); AG (*n* = 112); GG (*n* = 23). * *p*-value obtained from the multivariable GLM including the pre-miR-27a rs895819 polymorphism as additive.

The association of the pre-miR-27a rs895819 polymorphism with alcohol intake was especially relevant in the subgroup of drinking males (Figure 1).

Supplementary Figure S2 (Figure S2) shows the means (and SE) of alcohol consumption in males (A) and in females (B) (both including drinkers and non-drinkers), depending on the pre-miR-27a rs895819 polymorphism. Similar associations were found. Although the association between the pre-miR-27a rs895819 polymorphism and alcohol intake was tested in the codominant model (including the three genotypes), homozygous subjects for the variant allele (GG) presented higher means of alcohol consumption, supporting a recessive effect. This recessive effect was also confirmed later in the categorical analysis of drinking categories.

When alcoholic beverages were analyzed, we detected higher consumptions for wine (including red and white wine), beer and spirits (whisky, vodka, gin, rum, liquors, etc.) in GG subjects from the whole population, suggesting non-specificity for the pre-miR-27a rs895819 association with alcoholic beverages. However, differences among genotypes in the multivariable adjusted models only remained statistically significant for wine (*p* = 0.043). For beer, although in the unadjusted model, statistically-significant differences were detected (*p* = 0.041), this association did not reach the significance level after the multivariable adjustment. The association with spirits had a similar trend, but did not reach statistical significance. Therefore, factors related to statistical power may explain the difference in the statistical significance of the association between the pre-miR-27a rs895819 polymorphism and wine or beer, rather than arriving at the conclusion of a specific association of the SNP with wine intake. Thus, when we separately analyzed red wine and white wine (Supplementary Figure S3), we observed the same trend for the association (higher intake of red wine or of white wine in GG subjects), but the *p*-values were borderline significant for those wines.

### 2.2. Association between the Pre-miR-27a rs895819 Polymorphism and Drinking Categories

To minimize the influence of dealing with the potential limitations of the continuous variables of alcohol consumption, we subsequently considered categorical variables for alcohol intake. Thus, three groups of alcohol consumption were defined according to the reported daily intake of alcohol and sex-specific cut-off points defined at the international level [40,41,42]. This classification has been previously used by us in PREDIMED [42,43]. The categories were as follows (see the Methods for details): (1) no intake (0 g/day); (2) moderate alcohol intake (≤26.4 g/day for men and ≤13.2 g/day for women); and (3) high intake (>26.4 g/day for men and >13.2 g/day for women), corresponding to one “typical drink” (12 g of ethanol)/day for women and two drinks/day for men (i.e., exceeding recommended daily moderate drinking limits) [40,41,42,43,44,45]. In this population, 55.2% of women and 23.1% of men were non-drinkers. Conversely, 3.6% of women and 14.8% of men (7.5% of the population) consumed more than the sex-specific moderate recommendation (classified as having a high intake). We found a strong association between the pre-miR-27a rs895819 polymorphism and these categories of drinking (Table 3). For the whole population, the *p*-value for the association between the polymorphism and drinking categories was statistically significant (*p* = 0.005). Considering the population as a whole, the prevalence of a high alcohol intake was 17.5% in subjects with the GG genotype, whereas it was only 5.9% in AA subjects (*p* < 0.05). This association was seen both in men (30.0% of high drinkers among GG subjects versus 11.4% among AA subjects; *p* = 0.024) and in women (10.7% of high drinkers among GG subjects versus 2.7% among AA subjects; *p* = 0.010).

Non-drinkers and moderate-drinkers were further grouped, and we estimated the association between the polymorphism and high alcohol consumption.

In Table 4 we estimated the risk (by calculating the odds ratio (OR) and the 95% CI) of being a high alcohol drinker depending on the pre-miR-27a rs895819 polymorphism in the whole population and in men and women separately, after adjustment for potential confounders (see the Methods in Section 4.4). The AA genotype was considered the reference category, and we estimated the OR of being a high alcohol drinker (versus moderate and non-drinkers grouped) for the AG genotype and for the GG genotype. We found statistically-significant and highly consistent results in both men and women. For the population as a whole, subjects with the GG genotype were more likely than subjects with the AA genotype (OR: 3.84; 95% CI: 1.83–8.04, *p* < 0.001) to be high alcohol drinkers even after multivariate adjustment for potential confounders. This is a strong association considering the magnitude of the OR. Moreover, in women, we see approximately the same pattern of results as in men. For both, the associations seem to follow a recessive model as no statistically-significant differences were detected in AG individuals. Therefore, we merged AA + AG subjects in the same category for further sensitivity analyses.

### 2.3. Sensitivity Analysis of the Association between the Pre-miR-27a rs895819 Polymorphism and Drinking

Finally, we also performed a sensitivity analysis in order to estimate the magnitude of the association between the pre-miR-27a rs895819 polymorphism and drinking in relevant subgroups (sex, obesity, adherence to the Mediterranean diet (MedDiet), type 2 diabetes and hypertension) to analyze the homogeneity or heterogeneity of the associations. For this analysis, we used a dichotomous variable both for drinking (high drinker versus moderate + non-drinker) and for the polymorphism (AA + AG versus GG). We also estimated the interaction terms between the corresponding subgroup analyzed (sex, obesity, etc.) and the pre-miR-27a rs895819 polymorphism in determining the risk of being a high drinker to test the statistical significance of the heterogeneity of the associations in the corresponding strata. Table 5 shows sensitivity analysis estimations by sex, obesity, adherence to the MedDiet, type 2 diabetes and hypertension. We have detected a highly homogeneous effect (*p* for interactions >0.05 for all of the variables considered) in the association between the pre-miR-27a rs895819 polymorphism and alcohol drinking. The highest homogeneity was detected for obesity status in such a way that subjects with the GG genotype were more likely than subjects with the (AA + AG) genotype of being a high drinker, nearly with the same magnitude in both non-obese (OR: 3.31; 95% CI: 1.34–8.18, *p* = 0.01) and in obese subjects (OR: 3.87; 95% CI: 1.21–12.35, *p* = 0.022). Conversely, higher heterogeneity per type 2 diabetes status was observed (although without the interaction term being statistically significant), in such a way that the association between the pre-miR-27a polymorphism and high alcohol intake was attenuated in type 2 diabetic patients.

## 3. Discussion

This study has found a strong association between the functional polymorphism (rs895819) in the pre-miR-27a gene, consisting of a change from A>G, and total alcohol intake in an elderly Mediterranean population. Although several previous studies on humans have reported an influence of the miRNAs on alcohol consumption [16,21,24,27,28,29,46,47], those studies have mainly focused on measuring the expression of certain miRNAs in different tissues rather than on analyzing the influence of genetic polymorphisms in the genes that encode the miRNAs [29]. Pioneering studies measured the differential expression of the miRNA profile in the brain of alcoholics and compared this with the miRNA profiles in non-alcoholic controls, finding significant differences in the expression of various miRNAs [27,28], so suggesting an important regulatory role of the miRNAs in alcohol consumption. Specifically, Lewohl et al. [27], in 2011, undertook a study to analyze the differences in the profile of miRNA expression in the frontal cortex of 14 alcoholics and 13 age- and sex-matched controls. The alcoholics consumed more than 80 g of ethanol per day for most of their adult lives. Controls were defined as low alcohol consumption individuals (less than 20 g per day on average). They found significant differences in approximately 48 miRNAs. All were upregulated in the frontal cortex of alcoholics with a fold change of between 16% and 72%. The five miRNAs that showed the greatest differences of expression in the frontal cortexes between alcoholics and non-alcoholics were: miR-553, miR-369-3p, miR-18a, miR-339-5p and miR-1. The miR27a, which we have focused on in this study, did not appear in the list of the 48 differentially-expressed miRNAs. However, in our study, we are comparing a moderate alcohol intake, and Lewohl et al. [27] analyzed the effect of large amounts of ethanol. The authors concluded that the miRNAs could play an important role in the development of alcohol-related changes in the human brain, suggesting that the upregulation of miRNAs in the frontal cortex of human alcoholics may contribute to the deterioration and concomitant adaptation of neuronal functioning observed in individuals who abuse alcohol.

Later, Manzardo et al. [28] analyzed the differences of expression of the miRNAs isolated from the frontal cortex of nine alcoholics and nine matched controls. The authors also found statistical differences in the profile of miRNA expressed in cases and controls. However, although they found several upregulated miRNAs in alcoholics, outstanding among which were the miR-375, miR-29b, miR-377 and miR-379, the top-ranked miRNA did not overlap between both studies [27]. Despite the very important preliminary information that these studies provide us with, a comparison between these and later studies is not always easy, as different controls and different arrays are used. Additionally, in both studies, the controls were not abstemious individuals, but with moderate alcohol consumption that perhaps does not separate the differences well enough. Later studies have focused on the profiles of miRNAs circulating in plasma/serum as biomarkers of alcohol intake [28,47], also using different inclusion criteria for the analyzed individuals. Here again, the results are not very consistent, so emphasizing the need for greater standardization in defining cases, controls, the array used and a more direct measurement of the amount of alcohol consumed.

Faced with the current difficulties due to the lack of standardization when measuring miRNA expression, the results of which can also be different depending on the method and the tissue used for the measurement (brain, blood, etc.), the analysis of polymorphisms in genes encoding miRNAs is another interesting approach to investigating the role of miRNAs on alcohol consumption. This approach could be much more reproducible, given that the presence or absence of an SNP in the genes encoding an miRNA does not change whether the DNA comes from leukocytes or any other type of cell. Moreover, it is known that a single miRNA can target hundreds of mRNA transcripts for either translation repression or degradation, and the detection of a polymorphism in a gene encoding a particular miRNA can affect many mRNAs and have a great influence [48,49]; such as an intermediate hairpin precursor miRNA (pre-miRNA), which is transported to the cytoplasm by exportin-5 and further processed by another RNase III–like enzyme, Dicer, to the mature miRNA (for a review, see Kim, 2005).

miRNAs are initially transcribed as primary miRNAs (pri-miRNA). This long pri-miRNA (having several hundred nucleotides) is further processed into an intermediate hairpin precursor miRNA (pre-miRNA). The pre-miRNAs is further processed by Dicer, to the mature miRNAs [30,48]. Pre-miRNA polymorphisms may have an important functional role for miRNA binding and posttranscriptional regulation [49]. Moreover, genetic variation has been reported in pre-miRNAs to be relatively rare (only ten percent of human pre-miRNAs have identified SNPs [30]), so highlighting their functional relevance.

Despite this functional importance, there have been very few studies that have focused on studying SNPs in genes encoding microRNAs so as to analyze their relationship with alcohol intake. As far as we know, only one previously-published study has examined the association between a polymorphism in a miRNA gene and alcohol, in this case for alcohol-related disorders [29]. In that work, Novo-Veleiro et al. [29], using a case-control study, analyzed differences in prevalence for the miR-146a G>C (rs2910164) polymorphism in 301 male patients with alcohol-related disorders and 156 sex-matched healthy volunteers and reported a significantly higher prevalence of C-allele carriers (47.8%) among patients when compared to controls (35.9%). In that study, the total consumption of alcohol intake was not measured, nor were the different types of alcoholic drinks. Therefore, although Novo-Veleiro et al. [29] have indeed been the first to report a significant association between the rs2910164 polymorphism in a gene encoding an miRNA (the miR-146a G>C) and alcohol related disorders, we can claim that our study is the first that has shown an association between a polymorphism in a gene encoding an miRNA (in this case the miRNA27a) and daily alcohol consumption (in g/day), as well as the with alcoholic beverages, measured by a validated questionnaire at the population level including both men and women. Unfortunately, we do not have the miR-146a G>C (rs2910164) genotyped in our population to compare the results. Both investigations contribute to providing new knowledge regarding the potential role of miRNA polymorphisms in alcohol consumption.

Moreover, in our study, on classifying individuals as non-consumers, moderate consumers and high consumers of alcohol, the pre-miR-27a polymorphism (rs895819) was strongly associated with high alcohol consumption, fundamentally in a recessive way in individuals who present the two variant alleles (GG), even after adjustment for potential confounders. Additionally, we carried out a sensitivity analysis to test the homogeneity of this association depending on relevant variables, and we found a strong homogeneity across the different strata, so supporting the consistency of our findings. Thus, subjects with the GG genotype were more likely (approximately three times) than subjects with the AA genotype to report drinking more than the moderate daily limits for both men and women, in obese and in non-obese and in having or not a high adherence to the MedDiet pattern. For type 2 diabetic subjects, although we did not detect a statistically-significant interaction term with type 2 diabetes in the sensitivity analysis, the association was attenuated. This could reflect an environmental modification of the genetic influence. Thus, one of the recommendations that is usually made to type 2 diabetic subjects by health staff is that they reduce their alcoholic beverage intake. Hence, in the PREDIMED study, we have observed that alcohol intake in type 2 diabetic subjects is lower than in non-diabetics at baseline (mean ± SE: 4.6 ± 0.49 g/day versus 6.8 ± 0.42 g/day, respectively, *p* < 0.001; as well as the % of subjects having a high alcohol consumption: 4.3% versus 10.4%; *p* < 0.001). We may consider that the recommendation to reduce alcohol consumption could be an environmental factor that can modulate genetic susceptibility (in this case, for subjects having the GG genotype in the pre-miR27a polymorphism).

As this is the first time that an association between the pre-miR-27a A>G polymorphism (rs895819) and alcohol consumption has been found and also being an epidemiological study on humans, we do not know the mechanisms through which this association can take place. There are previous studies that have consistently shown that this polymorphism is functional, and the variant G allele is associated with higher levels of miR27a [34,35]. Likewise, there are also several previous studies that have associated this polymorphism with a greater risk of cancer [34,35,36,50,51]. Interestingly, many of those cancers are related to alcohol consumption (gastric, colorectal, lung, etc.) and perhaps this pre-miR-27a polymorphism could be acting as an indirect indicator of the amount of alcohol consumed, being greater in carriers of the variant G-allele, associated with a higher cancer risk, that being an example of a Mendelian randomization approach [52]. Mendelian randomization use genetic variants (mainly SNPs) as instrumental variables for exposures in association studies between the exposure (using the SNP as proxy for the exposure) and the outcome. [52]. Therefore, if the association between the pre-miR-27a-rs895819 polymorphism and alcohol intake is confirmed in further studies, this SNP could be used as an instrumental variable (proxy or indicator) acting as a genetic biomarker of alcohol intake in Mendelian randomization studies, in the same way as the SNPs in the alcohol-metabolizing enzymes, *ADH1B* (alcohol dehydrogenase 1B, Class I) or ALDH2 (aldehyde dehydrogenase 2 family), are used as instrumental variables for Mendelian randomization studies on cancer [52,53].

The strength of our study is that we have measured the habitual alcohol intake and the different types of alcoholic beverages consumed with a previously-validated questionnaire that ensures good validity and reliability, in an elderly population sample with moderate alcohol intake throughout the week. This population forms part of a study in which other variables are measured, and we, therefore, have a well-characterized population as far as the presence of other diseases, biochemical data, dietary data, exercise, etc., are concerned, all of which allows us to control the possible confounding factors. As a limitation, we should point out that, despite data on multiple diseases having been obtained, we did not specifically ask about alcoholic liver disease, which could reflect an indirect association with alcohol intake. However, bearing in mind that the alcohol intake of this population is moderate to low and that alcoholics were excluded, the prevalence of that disease in the population would be very low. This assumption can be supported by Table S1, showing liver enzyme activities and mean corpuscular volume by drinking categories in a random sample of participants in this study. Means of these biomarkers of potential alcohol damage are in general low. Another possible limitation is the generalization of these results to other populations with different characteristics of age and alcohol intake patterns, but the publication of these results will contribute to the undertaking of new studies and meta-analyses so as to compare that generalization.

The mechanisms through which the rs895819 in the pre-miR27a may influence alcohol consumption would also have to be investigated in greater depth, as well as how it affects the target mRNAs. In terms of target mRNAs for the miR-27a, there are several candidate genes that require investigation in greater depth. However, the fact that there have been previous studies [34,35] that have shown that the polymorphism is functional and can change the structure of the microRNA and its binding capability, with an increased miR-27a expression being detected in G-allele carriers compared to AA, allows us to argue that this polymorphism can alter the normal interaction between the miR27a and certain targets involved in regulating alcohol intake. Specifically, it is known that one of the targets of the miR27 is the serpin peptidase inhibitor clade I (Serpini1) [33], a protein primarily secreted by axons in the brain and known to be involved in the development of analgesic tolerance and, specifically, with morphine tolerance. Serpini1 knockout mice developed less analgesic tolerance than wild-type mice, supporting a role for miR27a and Serpini1 in the response to chronic opioid consumption [33]. Similarly, it is known that the effects of alcohol are mediated through intricate interactions between multiple neurochemical systems, including the opioid system [7]. Based on that, a closer binding between the miR27a and its target would make it less tolerant to alcohol (when the SNP is not present), whereas a looser binding between miR27a and its target (when the variant allele is present) may make it more tolerant to alcohol and necessary to drink more, as observed in homozygous subjects for the variant allele for this polymorphism. This potential mechanism is totally speculative, and more additional work is needed to support it.

The results of our work have been obtained from an elderly Mediterranean population with moderate, habitual alcohol consumption throughout the week. The generalization of the association of the pre-miR-27a rs895819 polymorphism with alcohol intake in different populations from the one studied (young people, greater alcohol consumers, high weekend alcohol consumers, etc.) needs to be established in future studies. However, the fact that this association is detected both in men and in women and that it has no statistically-significant heterogeneity by sex, obesity or degree of adherence to the Mediterranean diet allows us to hypothesize that its generalization for other populations may be high, although that would have to be checked in further studies.

## 4. Materials and Methods

### 4.1. Subjects

We analyzed 1007 participants (368 men and 639 women) in the PREDIMED (PREvención with DIeta MEDiterránea) trial recruited in the Valencia field center for whom DNA was available, the pre-miR-27a determined and valid data on alcohol intake obtained (See Flowchart in Figure S1). The PREDIMED is a multi-center, randomized, controlled clinical trial (controlled-trials.com number, ISRCTN35739639; ethics approval by the Institutional Review Board of the Hospital Clinic at Barcelona, Spain, 16/07/2002, under the protocol number “G03/140”) aimed at assessing the effects of the Mediterranean diet on the primary prevention of cardiovascular diseases [37]. The PREDIMED-Valencia field center, located on the East Mediterranean coast of Spain, was the field center that recruited the highest number of PREDIMED participants (*n* = 1094). Genotyping of the pre-miR-27a rs895819 polymorphism was carried out on 1042 participants with high quality DNA available. The 1007 participants with successful genotyping and alcohol data included in this analysis did not differ in the main characteristics from those of the total PREDIMED-Valencia cohort. PREDIMED eligible subjects were community-dwelling people (55–80 years of age for men; 60–80 years of age for women) who fulfilled at least one of two criteria: type 2 diabetes; 3 or more cardiovascular risk factors: current smoking, hypertension (blood pressure ≥140/90 mmHg or treatment with antihypertensive drugs), low-density lipoprotein cholesterol (LDL-C) ≥160 mg/dL (or treatment with hypolipidemic drugs), high-density lipoprotein cholesterol (HDL-C) ≤40 mg/dL, body mass index (BMI) ≥25 kg/m^2^ or a family history of premature cardiovascular diseases. Exclusion criteria included a personal history of cardiovascular disease, any severe chronic illness and drug or alcohol addiction [54]. For exclusion, in addition to the medical records known by the doctors, all of the participants were given the CAGE questionnaire (which is an acronym of its four questions: Cutting down, Annoyance by criticism, Guilty feeling, and Eye-openers) [37] on possible alcohol addiction. Out of the 4 questions in the questionnaire, a positive response to 2 was the reason for exclusion in accordance with the protocol for that questionnaire. None of the patients included in the PREDIMED-Valencia study had a positive response to 2 or more items in the CAGE questionnaire. The Institutional Review Board of the Valencia University (protocol number “G03/140”, 22 May 2003) approved the study protocol, and all participants provided written informed consent. In this report, we present data of the cross-sectional analysis at baseline.

### 4.2. Demographic, Clinical, Anthropometric, Dietary and Other Lifestyles Measurements

The baseline examination included the assessment of standard cardiovascular risk factors, medication use, socio-demographic factors and lifestyle variables, by validated questionnaires previously detailed [37]. These variables, as well as anthropometric variables, were used as covariates to adjust for potential confounding in the multivariate regression models. Weight and height were measured with light clothing and no shoes with calibrated scales and a wall-mounted stadiometer, respectively. BMI was calculated as the weight (in kg) divided by the height (in m^2^). Obesity was defined as a BMI ≥ 30 kg/m^2^ and in accordance with World Health Organization (WHO, Geneva, Switzerland) criteria. Blood pressure was measured by trained personnel using a validated semi-automatic oscillometer (Omron HEM-70CP; Hoofddrop, The Netherlands) with the subject seated as previously reported [37].

The level of adherence to the Mediterranean diet was measured by a validated 14-item questionnaire, and subjects were classified as having low or high adherence, based on the population mean (9 points) [55]. Food consumption was determined by a validated 137-item semi-quantitative FFQ [39]. This FFQ, which also included nine questions on consumption of different alcoholic beverages (different types of wine, beer and spirits), was used to measure the intake of beverages with alcohol. In this FFQ, both for foods and drinks, there were questions about the average intake for each item over the previous year, including the baseline visit, which is when that questionnaire was administered. For each alcoholic beverage included in the questionnaire (vintage red wine, young red wine, young rosé, white wine, cava, beers and spirits, including whisky, gin, rum, vodka and liqueurs), questions were asked for a typical Spanish measure of the same (as detailed in our previous publication [39]). Alcohol intake (g/day) was calculated by multiplying the amount of the beverage (mL) by the respective degree (% alcohol) and the constant 0.80 to transform alcohol volumes into weight. The sum of all of that is the total amount of alcohol in g/day consumed for each person. Abstainers were those individuals for whom the sum was zero grams per day. For the analysis of specific beverages, total wine, total beer and total spirits were considered as grouped variables. Further, red wine (including vintage red wine, young red wine and young rosé) and white wine were also analyzed.

This FFQ was previously validated by us [39] in a similar population through a standard validation procedure in the following way: The FFQ was administered twice (FFQ1 and FFQ2) to explore reproducibility at 1 year. Four 3-d dietary records (DR) were used as a reference to explore validity; participants therefore recorded their food intake over 12 days in the course of 1 year. The intra-class correlation coefficient between alcohol intake from the FFQ and repeated DR was 0.82 [39]. This high correlation coefficient value allows us to conclude that the FFQ measurements have good reproducibility and a relative validity similar to those of FFQs used in other prospective studies and that we, therefore, have a good instrument for measuring habitual alcohol and alcoholic beverage intake.

Once the amount of alcohol consumed was calculated (in g/day as the annual average) for each individual on the basis of the type and amount of alcoholic beverages consumed, we undertook an additional classification into alcohol intake categories. Although there are several cut-off points to classify alcohol intake, we used international criteria of alcohol intake [40,41,42], as we did in previous studies [42,43], with the aim of ensuring the best comparability between publications. Thus, three groups of alcohol consumption were defined according to the reported daily intake of alcohol: no intake (0 g/day), moderate intake (≤26.4 g/day for men and ≤13.2 g/day for women) and high intake (>26.4 g/day for men and >13.2 g/day for women). These gram amounts correspond to 1 drink/day for women and 2 drinks/day for men [41,42,43]. The cut-off points were chosen to facilitate the interpretation of the gram-based alcohol categories and according to recommended upper limits of daily alcohol consumption of one drink/day for women and two drinks/day for men stated by the several organizations and frequently used in epidemiological studies on alcohol intake [41,42,43,44,45].

Physical activity was estimated by the validated Minnesota Leisure-Time Physical Activity questionnaire, as previously reported [37].

### 4.3. Biochemical Determinations, DNA Extraction and Genotyping

Fasting blood samples were obtained for each participant and stored at −80 °C until biochemical analyses. Fasting glucose, total cholesterol, triglycerides, HDL-C and LDL-C were determined as previously reported [56]. In a random sample of participants (detailed in Table S1), liver enzymes and mean corpuscular volume were also determined at baseline by standard procedures. LDL-C concentrations were estimated with the equation of Friedewald et al. whenever triglycerides were <400 mg/dL. Genomic DNA was extracted from buffy coat with the MagNaPure LC DNA Isolation kit (ROCHE Diagnostics, Indianapolis, IN, USA). The pre-miR-27a rs895819 A>G polymorphism was genotyped using the HumanOmniExpress Illumina BeadChip (Illumina, San Diego, CA, USA) by standard techniques. Genotype frequencies were consistent with Hardy–Weinberg equilibrium (*p* = 0.111).

### 4.4. Statistical Analyses

Chi-square tests were used to compare proportions. Triglyceride concentrations were log-transformed for the statistical analyses. Alcohol intake was square root transformed for the statistical analyses. In both cases, untransformed values were presented for means and standard error, but the *p*-values were obtained with the square root-transformed variables. ANOVA tests were applied to compare crude means according to the pre-miR-27a rs895819 genotypes. Co-dominant and recessive models of inheritance were first tested to know the effects of the variant allele. In the analysis for the additive model, the pre-miR-27a rs895819 A>G polymorphism was considered as a linear term coded as 0, 1 or 2 depending on the number of G-alleles, with the homozygote wild-type coded as 0. Taking into account that similar effects were detected in AA and AG subjects, a recessive model GG versus (AA + AG) was also considered. Results were presented both for the co-dominant/additive and for the recessive models for the whole population. In addition, a stratified analysis of total alcohol intake in male drinkers, men (drinkers and non-drinkers) and women (drinkers and non-drinkers) according to the pre-miR-27a rs895819 polymorphism was undertaken.

Unadjusted (crude) and multivariable adjusted general linear models (GLM) were used for continuous variables, and logistic regression models were used for dichotomous variables (categories of alcohol consumption). Models were adjusted for potential confounders including age, sex, type 2 diabetes, obesity, hypertension, dyslipidemia, physical activity, smoking and total energy intake. Odds ratios (OR) and 95% confidence intervals (CI) for the risk of being in the category of high alcohol consumption were estimated, and a multivariable logistic regression was performed. Analyses were done for the whole population and stratified by sex when indicated. A sensitivity analysis to test the homogeneity of the pre-miR-27a rs895819 polymorphism (as recessive) with the risk of having a high drinking pattern was carried out taking into account the following categories: sex, obesity, type 2 diabetes, hypertension and adherence to Mediterranean Diet. Interaction terms between the pre-miR-27a rs895819 polymorphism (recessive) and the corresponding variables for stratification were calculated in the corresponding hierarchical regression model (logistic). The stratified estimations of the associations were also carried out with the multivariate adjusted regression models. Statistical analyses were performed with the IBM SPSS Statistics Version 21.0 (IBM, Armonk, NY, USA). All tests were two-tailed, and *p*-values <0.05 were considered statistically significant.

## 5. Conclusions

In conclusion, in this study, we have reported, for the very first time, a significant association between an SNP in a miRNA (rs895819 A>G in the pre-miR27a) and total alcohol consumption (higher in homozygous subjects for the variant G-allele) and specific beverages, so providing more information on the importance of miRNAs in regulating alcohol intake, not only through epigenetic mechanisms, but also through the genetic variants in their DNA sequence, which has to be taken into account in later omic integration studies analyzing the miRNAs’ regulome. The generalization of the association of the pre-miR-27a rs895819 polymorphism with alcohol intake in different populations from the one studied needs to be established in future studies.

## Figures and Tables

**Table 1:** Demographic, clinical and lifestyle characteristics of the study participants at baseline according to the pre-miR-27a-rs895819 A>G polymorphism ^1^.

			pre-miR-27a-rs895819 A>G genotypes						
Variable	Total (*n* = 1007)		AA (*n* = 540)		AG (*n* = 381)		GG (*n* = 86)		*p* ^2^
Male sex: *n*, %	368	(36.5%)	201	(37.2%)	137	(36.0%)	30	(34.9%)	0.876
Type 2 diabetes: *n*, % ^3^	468	(46.5%)	243	(45.0%)	188	(49.3%)	37	(43.0%)	0.342
Hypertension: *n*, % ^4^	844	(83.8%)	451	(83.5%)	320	(84.0%)	73	(84.9%)	0.944
Dyslipidemia: *n*, %	769	(76.4%)	408	(75.6%)	290	(76.1%)	71	(82.6%)	0.361
Obesity: *n*, % ^5^	512	(50.8%)	269	(49.8%)	199	(52.2%)	44	(51.2%)	0.769
*Smokers: n*, *%*	–	–	–	–	–	–	–	–	0.504
Current	127	(12.6%)	67	(12.4%)	44	(11.5%)	16	(18.6%)	–
Former	234	(23.2%)	128	(23.7%)	88	(23.1%)	18	(20.9%)	–
Never	646	(64.2%)	345	(63.9%)	249	(65.4%)	52	(60.5%)	–
Age (years)	66.8	(0.2)	67.0	(0.3)	66.9	(0.3)	66.1	(0.7)	0.467
Weight (kg)	77.2	(0.4)	77.2	(0.5)	77.2	(0.6)	77.1	(1.4)	0.997
BMI (kg/m^2^)	30.6	(0.1)	30.7	(0.2)	30.7	(0.2)	30.4	(0.5)	0.842
Waist circumference (cm)	103.0	(0.4)	103.0	(0.5)	103.6	(0.6)	102.2	(1.3)	0.553
SBP (mm Hg)	147.1	(0.7)	147.0	(0.9)	147.8	(1.1)	144.5	(2.2)	0.444
DBP (mm Hg)	82.0	(0.3)	82.1	(0.5)	82.3	(0.6)	79.5	(1.0)	0.092
Heart rate (bpm)	72.4	(0.3)	72.1	(0.4)	72.8	(0.6)	72.3	(1.1)	0.678
Total cholesterol (mg/dL)	208.1	(1.3)	208.1	(1.7)	207.6	(2.1)	210.7	(4.6)	0.806
LDL-C (mg/dL)	129.4	(1.1)	129.2	(1.5)	129.3	(1.9)	130.6	(4.0)	0.946
HDL-C (mg/dL)	52.6	(0.4)	52.7	(0.6)	52.3	(0.7)	54.0	(1.8)	0.568
Triglycerides (mg/dL)	131.5	(2.2)	133.3	(3.1)	129.6	(3.2)	129.4	(9.7)	0.674
Fasting glucose (mg/dL)	120.4	(1.3)	120.3	(1.8)	120.9	(2.0)	118.1	(3.6)	0.843
Energy intake (kcal/day)	2210	(20)	2198	(28)	2221	(31)	2238	(74)	0.780
Total fat (g/day)	95.1	(1.0)	95.5	(1.4)	94.8	(1.5)	93.7	(2.8)	0.852
Saturated fat (g/day)	25.1	(0.3)	25.1	(0.4)	25.4	(0.5)	23.7	(0.8)	0.337
MUFA (g/day)	46.4	(0.5)	46.7	(0.7)	46.1	(0.8)	46.1	(1.5)	0.812
PUFA (g/day)	15.6	(0.2)	15.7	(0.3)	15.6	(0.3)	15.2	(0.7)	0.856
Proteins (g/day)	92.8	(0.8)	92.7	(1.2)	92.6	(1.3)	93.5	(3.0)	0.962
Carbohydrates (g/day)	235.6	(2.6)	232.6	(3.5)	239.1	(4.2)	239.3	(10.9)	0.463
Adherence to the MedDiet (points) ^6^	8.4	(0.1)	8.5	(0.1)	8.4	(0.1)	8.8	(0.2)	0.246
Physical activity (METs-min/day)	169.8	(5.5)	169.6	(7.8)	173.1	(8.7)	156.3	(15.8)	0.721

^1^ Values are expressed as the mean (standard error) for continuous variables or as (*n*, %) for categorical variables. MUFA, monounsaturated fatty acids; PUFA, polyunsaturated fatty acids; MedDiet, Mediterranean diet; MET, Metabolic Equivalent of Task; ^2^ unadjusted *p*-value obtained in the ANOVA test; ^3^ Type 2 diabetes was defined as a fasting blood glucose level of 126 mg/dL or higher on two occasions, a 2-h plasma glucose level of 200 mg/dL or higher during a 75-g oral glucose-tolerance test or the use of antidiabetic medication; ^4^ Hypertension was defined as a systolic blood pressure (SPB) of 140 mm Hg or higher, a diastolic blood pressure (DBP) of 90 mm Hg or higher or the use of antihypertensive therapy; ^5^ Obesity was defined as Body Mass Index (BMI) greater or equal to 30 kg/m^2^; ^6^ Based on a 14-point screener of adherence.

**Table 2:** Association of the pre-miR-27a-rs895819 A>G polymorphism with total alcohol consumption and alcoholic beverages in the whole population ^1^.

Genotypes									
Alcoholic Beverage ^2,3^	Total (*n* = 1007)		AA (*n* = 540)		AG (*n* = 381)		GG (*n* = 86)		*p* ^4,5^
Total alcohol (g/day) ^2^	5.8	(0.3)	5.2	(4.4–6.0)	5.9	(4.8–6.9)	9.1	(5.6–12.6)	0.020
Total alcohol (g/day) ^3^	–	–	7.4	(6.3–8.6)	8.2	(6.9–9.5)	11.0	(8.2–13.1)	0.016
Total wine (mL/day) ^2^	36.8	(2.3)	34.8	(28.7–40.7)	35.7	(28.8–42.5)	54.9	(32.0–77.7)	0.036
Total wine (mL/day) ^3^	–	–	47.8	(38.8–56.1)	49.4	(39.9–58.8)	66.4	(50.9–82.0)	0.043
Total beer (mL/day) ^2^	39.9	(3.4)	35.1	(27.6–42.6)	38.8	(28.3–49.3)	75.5	(33.4–117.8)	0.041
Total beer (mL/day) ^3^	–	–	56.7	(39.5–65.8)	56.0	(41.6–70.4)	88.2	(64.6–111.7)	0.142
Total spirits (mL/day) ^2^	2.0	(0.3)	1.4	(0.8–1.9)	2.6	(0.9–3.8)	2.9	(0.9–5.0)	0.172
Total spirits (mL/day) ^3^	–	–	3.6	(1.4–3.6)	3.7	(2.5–4.9)	4.0	(2.0–6.0)	0.073

^1^ Values are expressed as the mean (and standard error) for the whole population and as the mean (and 95% confidence intervals: CI for genotype groups); ^2^ The first row presents the *p*-value of the association between the SNP and total alcohol/alcoholic beverage in the crude (unadjusted model). The square root transformed variables were used to test the statistical significance of the crude association. The *p*-value vas obtained as a linear trend for the genotype; ^3^ The second row presents the adjusted *p*-value of the association between the SNP and total alcohol/alcoholic beverage in the multivariable model adjusted for sex, age, type 2 diabetes, hypertension, dyslipidemia, obesity, smoking, physical activity and total energy intake in the general linear model (GML) for the corresponding square root transformed variables; ^4^ unadjusted means, SE and 95% CI for the corresponding untransformed variables for total alcohol and alcoholic beverages in the whole population and by genotypes; beverages; ^5^ Adjusted (for sex, age, type 2 diabetes, hypertension, dyslipidemia, obesity, smoking, physical activity and total energy intake) mean and 95% CI for total alcohol intake and alcoholic beverages by genotype. The polymorphism was tested for a linear trend.

**Table 3:** Association between the pre-miR-27a-rs895819 A>G polymorphism and drinker category in the whole population and stratified by sex ^1,2^.

Whole Population							Men				Women			
Alcohol Consumption	Non-Drinkers (0 g/Day)		Moderate (<26.4 g/Day for Men) (<13.2 g/Day for Women)		High (>26.4 g/Day for Men) (>13.2 g/Day for Women)		Non-Drinkers + Moderate		High		Non-Drinkers + Moderate		High	
**Genotypes**	(*n* = 540)		(*n* = 381)		(*n* = 86)		(*n* = 315)		(*n* = 53)		(*n* = 616)		(*n* = 23)	
***p*^3^ polymorphism**	0.005		–	–	–	–	0.024		–	–	0.010		–	–
**AA: *n* (%)**	244	(45.2%)	264	(48.9%)	32	(5.9%)	178	(88.6%)	23	(11.4%)	330	(97.3%)	9	(2.7%)
**AG: *n* (%)**	160	(42.0%)	192	(50.4%)	29	(7.6%)	116	(84.7%)	21	(15.3%)	236	(96.7%)	8	(3.3%)
**GG: *n* (%)**	34	(39.5%)	37	(43.0%)	15	(17.4%)	21	(70.0%)	9	(30.0%)	50	(89.3%)	6	(10.7%)

^1^ Values are expressed as *n* and %; ^2^ the three groups were defined according to the reported daily intake of alcohol: no intake (0 g/day), moderate intake (<26.4 g/day for men and <13.2 g/day for women) and high intake (>26.4 g/day for men and >13.2 g/day for women); ^3^ unadjusted *p*-values obtained in the chi square tests for the association between genotypes and drinking categories in the whole population or in men and women separately.

**Table 4:** Association between the pre-miR-27a-rs895819 A>G polymorphism and the risk of having a high alcohol intake in the whole population and stratified by sex ^1^.

Whole Population						
Polymorphism	Model 1			Model 2		
Genotypes	OR	95% CI	*p*	OR	95% CI	*p*
AA (*n* = 540)	1.00	(reference)	–	1.00	(reference)	–
AG (*n* = 381)	1.34	(0.79–2.29)	0.276	1.45	(0.81–2.53)	0.190
GG (*n* = 86)	3.57	(1.79–7.16)	<0.001	3.84	(1.83–8.04)	<0.001
**Men**						
**Polymorphism**	**Model 1**			**Model 2**		
**Genotypes**	**OR**	**95% CI**	***p***	**OR**	**95% CI**	***p***
AA (*n* = 201)	1.00	(reference)	–	1.00	(reference)	–
AG (*n* = 137)	1.40	(0.73–2.64)	0.311	1.52	(0.78–2.99)	0.220
GG (*n* = 30)	3.01	(1.22–7.45)	0.017	3.42	(1.28–9.11)	0.014
**Women**						
**Polymorphism**	**Model 1**			**Model 2**		
**Genotypes**	**OR**	**95% CI**	***p***	**OR**	**95% CI**	***p***
AA (*n* = 339)	1.00	(reference)	–	1.00	(reference)	–
AG (*n* = 244)	1.24	(0.47–3.27)	0.660	1.44	(0.52–3.96)	0.486
GG (*n* = 56)	4.39	(1.50–12.87)	0.007	4.61	(1.44–14.83)	0.010

^1^ OR and 95% CI were estimated by multivariable logistic regression models (high alcohol intake versus non-intake + moderate); Model 1: adjusted for sex and age; Model 2: additionally adjusted for type 2 diabetes, hypertension, dyslipidemia, obesity, smoking, physical activity and total energy intake.

**Table 5:** Sensitivity analysis of the association between the pre-miR-27a-rs895819 A>G polymorphism and risk of having a high alcohol intake ^1^.

Variable	% Drinker High ^2^			Risk ^3^		
Sex	AA + AG	GG	*p* ^4^	OR	95% CI	*p* ^5^
Men (*n* = 368)	13.0%	30.0%	0.011	2.84	(1.12–7.17)	0.028
Women (*n* = 639)	2.9%	10.7%	0.003	3.79	(1.36–11.64)	0.012
*p* ^6^ for interaction:	0.774					
**Variable**	**% Drinker High ^2^**			**Risk ^3^**		
Obesity	AA + AG	GG	*p* ^4^	OR	95% CI	*p* ^5^
Non-obese (*n* = 495)	8.2%	21.0%	0.005	3.31	(1.34–8.18)	0.010
Obese (*n* = 512)	5.1%	13.6%	0.022	3.87	(1.21–12.35)	0.022
*p* ^6^ for interaction:	0.934					
Adherence to MedDiet	AA + AG	GG	*p* ^4^	OR	95% CI	*p* ^5^
Low < 9 (*n* = 511)	5.1%	17.1%	0.002	4.56	(1.71–14.34)	0.003
High ≥ 9 (*n* = 496)	8.2%	17.8%	0.033	2.49	(0.09–6.60)	0.069
*p* ^6^ for interaction:	0.546					
**Variable**	**% Drinker High ^2^**			**Risk ^3^**		
Diabetes	AA + AG	GG	*p* ^4^	OR	95% CI	*p* ^5^
No (*n* = 539)	9.0%	24.5%	0.001	3.56	(1.54–8.23)	0.003
Yes (*n* = 468)	3.9%	8.1%	0.221	2.06	(0.52–8.18)	0.304
*p* ^6^ for interaction:	0.547					
**Variable**	**% Drinker High ^2^**			**Risk ^3^**		
Hypertension	AA + AG	GG	*p* ^4^	OR	95% CI	*p* ^5^
No (*n* = 163)	8.0%	23.1%	0.103	4.59	(0.77–27.59)	0.096
Yes (*n* = 844)	6.4%	16.4%	0.004	3.22	(1.50–6.90)	0.003
*p* ^6^ for interaction:	0.818					

^1^ OR and 95% CI were estimated by multivariable logistic regression models adjusted for the covariates indicated below; ^2^ % of subjects having a high alcohol intake (>26.4 g/day in men and >13.2 g/day in women) depending on the pre-miR-27a-rs895819 polymorphism; ^3^ OR of being a high alcohol drinker in comparison with non-drinker + moderate, depending on the variable considered for GG individuals versus AA + AG (recessive model); ^4^ unadjusted *p*-value for comparison of percentages; ^5^ model adjusted for sex, age, type 2 diabetes, hypertension, dyslipidemia, obesity, smoking, physical activity and total energy intake; ^6^ *p*-value for the interaction term between the corresponding variable (sex, obesity, adherence to MedDiet, diabetes or hypertension) and the pre-miR-27a-rs895819 polymorphism (recessive) in the multivariable adjusted model.

## Supplementary Materials

Supplementary materials can be found at www.mdpi.com/1422-0067/17/8/1338/s1.
