# Supplementary material for: MicroRNAs and Drinking: Association between the Pre-miR-27a rs895819 Polymorphism and Alcohol Consumption in a Mediterranean Population
Source: Int J Mol Sci. 2016 Aug 16;17(8):1338. doi: 10.3390/ijms17081338 (PMC5000735; doi:10.3390/ijms17081338)
Supplement: Supplementary file 1 [file ijms-17-01338-s001.pdf]

Supplementary Materials: MicroRNAs and Drinking: Association between the Pre-miR-27a rs895819 Polymorphism and Alcohol Consumption in a Mediterranean Population

Rocío Barragán, Oscar Coltell, Eva M. Asensio, Francesc Francés, José V. Sorlí, Ramon Estruch, Albert Salas-Huetos, Jose M. Ordovas and Dolores Corella

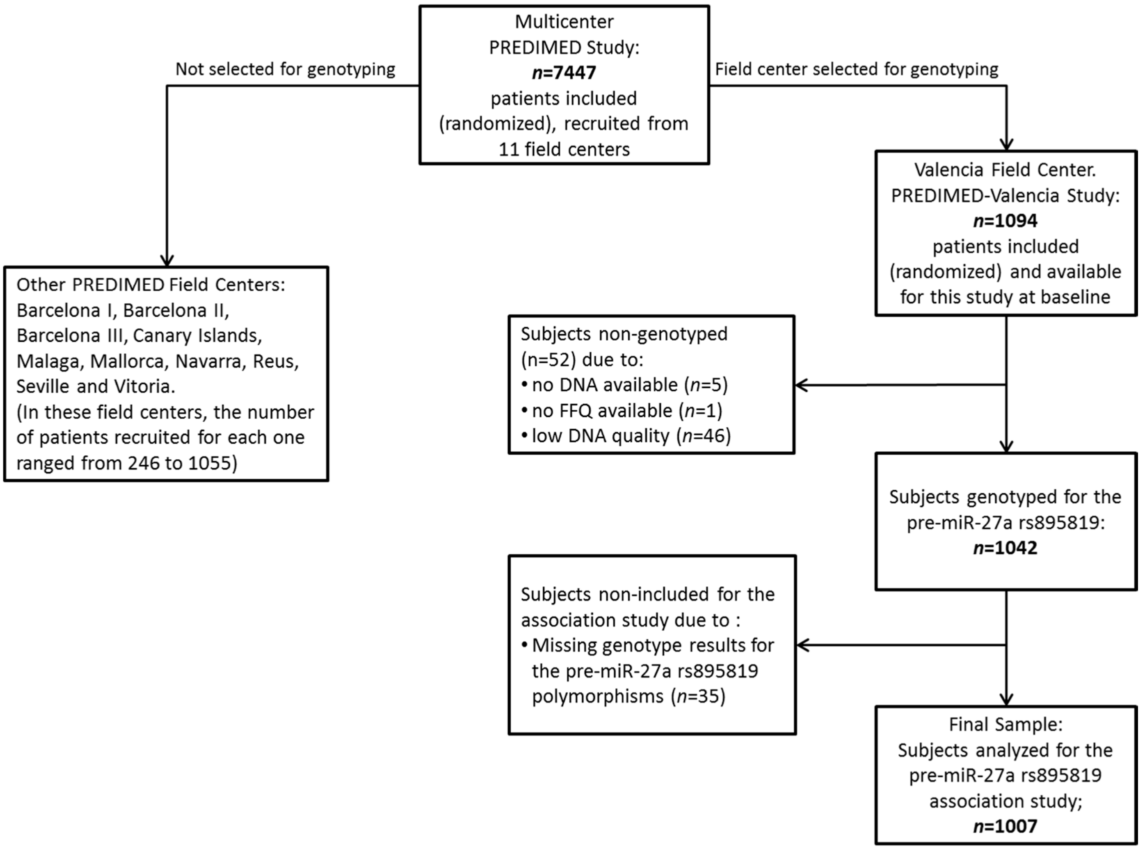

Figure S1. Flow-chart of the PREDIMED-Valencia Study.

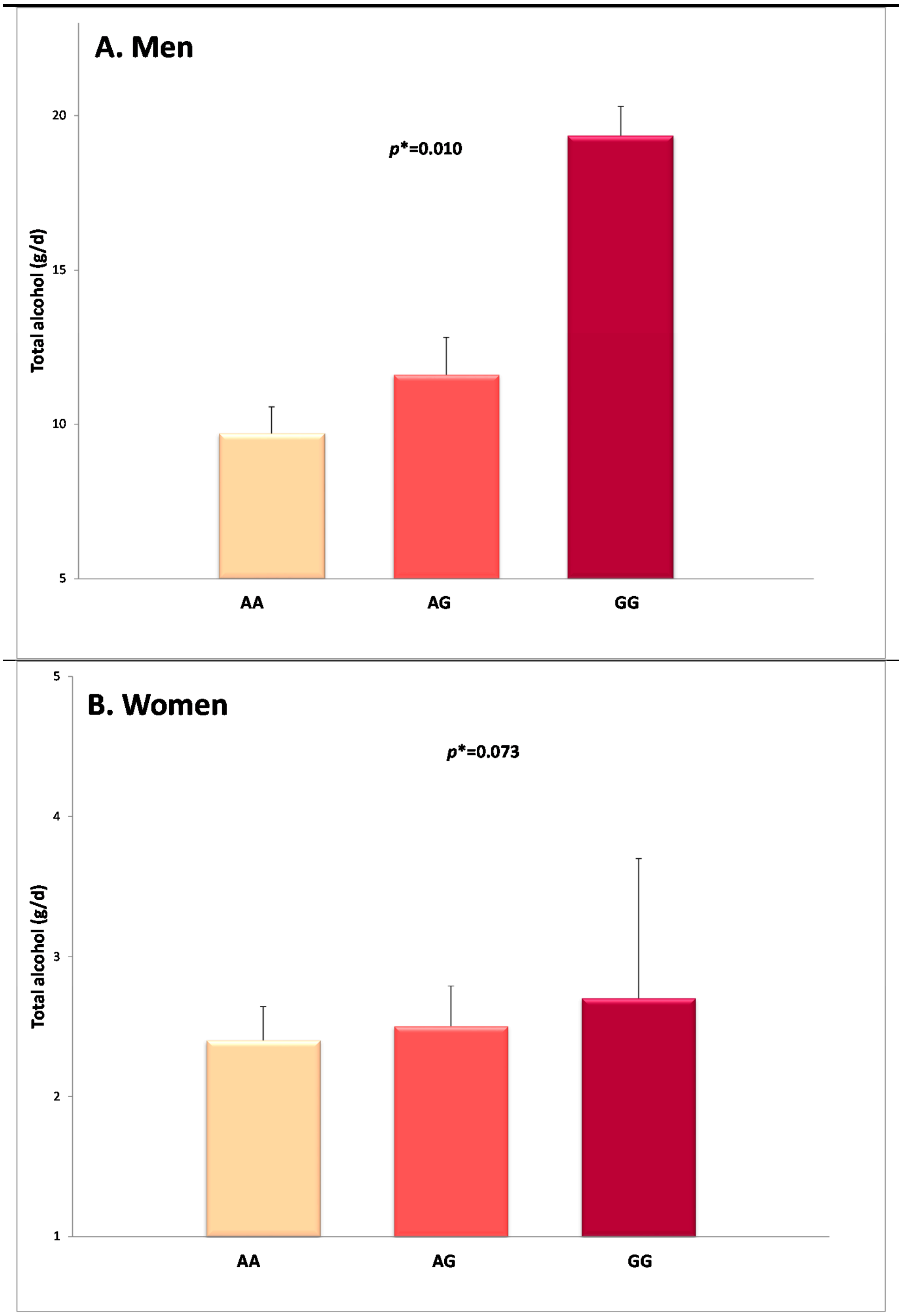

Figure S2. Total alcohol consumption (g/day) in men (**A**) and women (**B**). Means and standard errors (SE) of total alcohol intake (g/day), depending on the pre-miR-27a rs895819 polymorphism, are presented as untransformed variables (n = 368 for men, and n = 639 for women); p-values are calculated for the square root transformed variables; p* indicates the p-value for linear trend (additive model for the SNP). When these models were adjusted for age, type-2 diabetes, obesity, hypertension, dyslipidemia, physical activity, smoking and total energy intake, the adjusted p-values were p_{adj} = 0.034 for men, and p_{adj} = 0.292 for women.

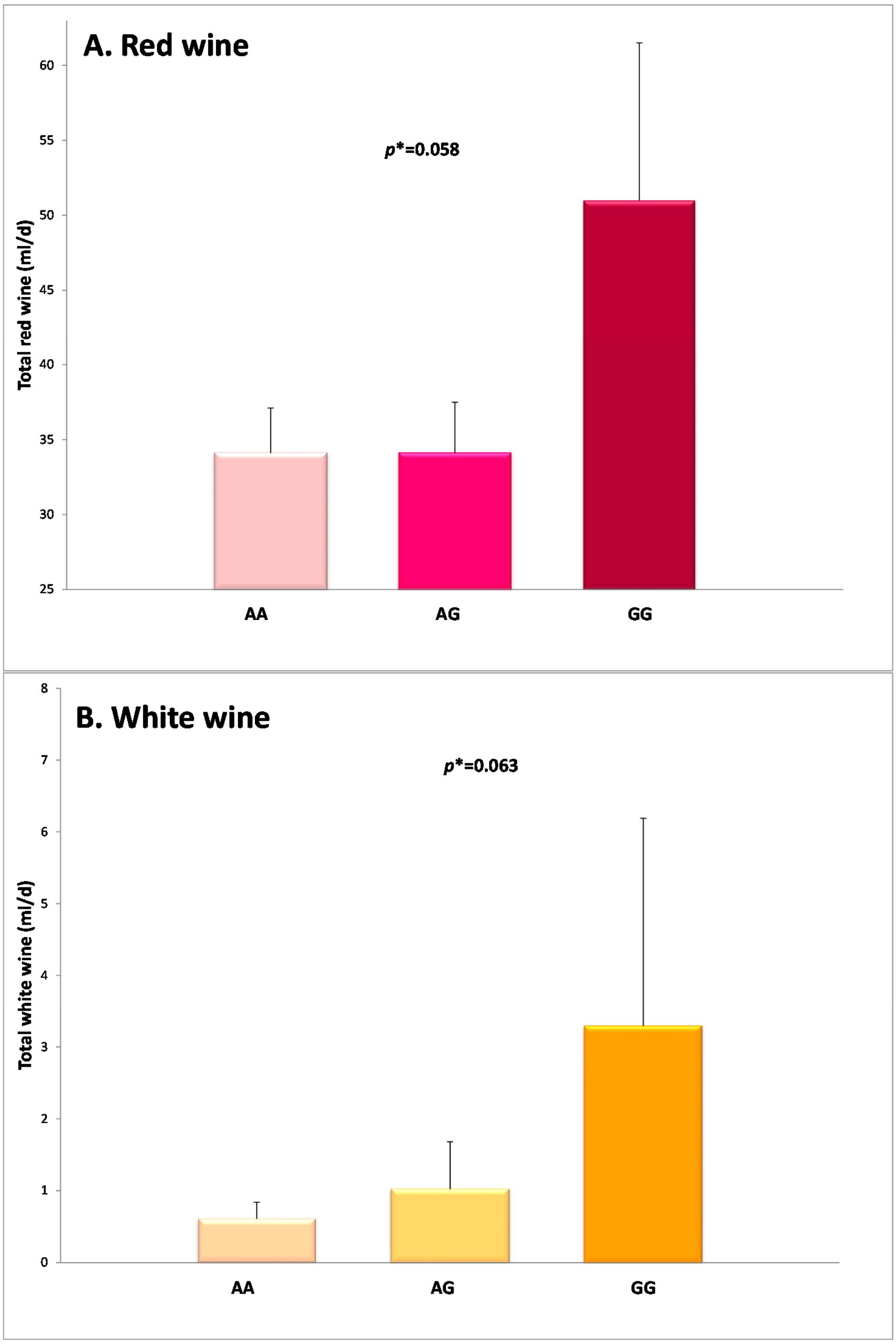

Figure S3. Consumption (mL/day) of red wine (**A**) and white wine (**B**) in both men and women. Means and standard errors (SE) of total consumption (mL/day) of red and white wine, depending on pre-miR-27a rs895819 polymorphism, are presented as untransformed variables (n = 1007 participants). p-values are calculated for the square root transformed variables. p^* indicates the p-value for linear trend (additive model for the SNP). When these models were adjusted for age, type 2 diabetes, obesity, hypertension, dyslipidemia, physical activity, smoking and total energy intake, the adjusted p-values were $p_{adj} = 0.165$ for men, and $p_{adj} = 0.210$ for women.

Table S1. Association between liver enzymes and mean corpuscular volume with alcohol consumption [1,2].

Liver Enzymes and Mean Corpuscular Volume [3]	Total (*n* =749)	Non-Drinker (*n* =329)	Moderate Drinker (*n* = 371)	High Drinker (*n* = 49)	p^4	p^5
AST (U/L) (*n* = 672)	21.86 (0.34)	21.60 (0.54)	21.99 (0.45)	22.80 (1.66)	0.424	0.793
ALT (U/L) (*n* = 749)	24.10 (0.45)	23.17 (0.63)	24.70 (0.69)	26.10 (1.46)	0.122	0.536
GGT (U/L) (*n* = 521)	28.07 (0.96)	25.23 (1.96)	29.69 (1.51)	22.78 (3.40)	0.052	0.288
MCV (fL/RBC) (*n* = 567)	89.48 (0.21)	88.80 (0.32)	89.70 (0.31)	92.01 (0.87)	0.001	0.045

[1]: Values are expressed as mean (standard error); [2]: Non-drinker: 0 g/day; Moderate drinker: ≤26.4 g/day for men and ≤13.2 g/day for women; High drinker: >26.4 g/day for men and >13.2 g/day for women; [3]: *n* values are the number of subjects having almost one liver enzyme determination (*n* = 749 for ALT). For the other parameters, the corresponding *n* has been indicated between brackets; [4]: *p* Unadjusted *p*-value obtained in the ANOVA test for linear trend; [5]: *p* Adjusted *p*-value for sex and age in the multivariable GML; AST: aspartate aminotransferase (old GOT); ALT: alanine aminotransferase (old GPT); GGT: gamma glutamyl transferase; and MCV: mean corpuscular volume. AST, ALT and GGT are expressed in units of enzymatic activity (the amount of enzyme that catalyzes the conversion of 1 micro mole of substrate per minute) in a volume of 1 liter. MCV is expressed in femtoLiters per Red Blood Cell size.
